# Supplementary material for: Quasi-3D Mechanistic Model for Predicting Eye Drop Distribution in the Human Tear Film
Source: Bioengineering (Basel). 2025 Jul 30;12(8):825. doi: 10.3390/bioengineering12080825 (PMC12383929; doi:10.3390/bioengineering12080825)
Supplement: Supplementary file 1 [file bioengineering-12-00825-s001.zip › bioengineering-3725131-supplementary.pdf]

Supplemental Material

Table S1. Qualitative comparison of Q3D, FEM, and PBPK (ODE-based) modeling strategies.

Parameter	Q3D	PBPK (ODE)	FEM
**Mathematical Foundation**	Uses partial differential equations (PDE)	Uses ordinary differential equations (ODE)	Uses partial differential equations (PDE)
**Model Development Complexity and Time**	Moderate: CoBi platform simplifies setup, minimal manual coding of equations	Low to High: simple for basic models, complex when discretization needed (100s of equations/loops)	High: complex mesh setup and implementation
**Computational Speed**	Moderate: faster than FEM, slightly slower than ODEs	Fast: fastest among the three approaches	Slow: most computationally intensive
**Computational Resources**	Moderate to low resource requirements	Low resource requirements	High resource requirements
**Mesh Setup**	Mesh setup is simple and user friendly	Not applicable (no spatial discretization)	Manual mesh setup is difficult and time-consuming
**Accuracy**	Good spatial resolution with quasi-3D approximations	Limited spatial resolution, suitable for lumped parameter models	Highest accuracy with full 3D spatial resolution
**Visualization Capabilities**	Strong visualization capabilities	Limited visualization (typically 1D plots)	Excellent 3D visualization capabilities
**Multiphysics Coupling**	Easier coupling compared to manual approaches, using CoBi	Manual coupling required - more complex to implement	Easier coupling within FEM framework
**Scalability**	Good scalability and works well for extended domains and layered tissues	Excellent for simple models, limited for spatially discretized problems	Limited scalability due to computational demands
**User Expertise Required**	Moderate: Requires some platform familiarity.	Low to High: Depends on model complexity	High: Requires FEM expertise and mesh generation skills
**Best Use Cases**	Problems with skewed aspect ratios (similar to 1D geometry), moderate complexity requiring spatial detail with computational efficiency	Simple lumped parameter models, rapid prototyping	Complex 3D geometries, high-accuracy applications requiring full 3D spatial resolution where FEM complexity is justified

Table S2. ODEs characterizing species transport between all ocular regions.

**Ocular Region**	**Species Transport Equation**
Upper Bulbar Conjunctiva (UB)	$V_{UB}\frac{dC_{UB}}{dt} = J_{UB-TF} + J_{UF-UB} + J_{UM-UB} - J_{UB-Elim}$
Upper Palpebral Conjunctiva (UP)	$V_{UP}\frac{dC_{PU}}{dt} = J_{UF-UP} - J_{UP-Elim}$
Upper Fornical Sac (UF)	$V_{UF}\frac{dC_{UF}}{dt} = \frac{dC_{diss,UF}}{dt} - J_{UM-UF} - J_{UF-UP} - J_{UF-UB} - \frac{dV_{UF}}{dt}C_{UF}$
Upper Meniscus (UM)	$V_{UM}\frac{dC_{UM}}{dt} = \frac{dC_{diss,UM}}{dt} + J_{UM-UF} - J_{UM-UB} - J_{UM-Drain} - \frac{dV_{UM}}{dt}C_{UM}$
Tear Film (TF)	$V_{TF}\frac{dC_{TF}}{dt} = \frac{dC_{diss,TF}}{dt} - J_{LB-TF} - J_{UB-TF} - J_{CorEpi-TF} - \frac{dV_{TF}}{dt}C_{TF}$
Lower Meniscus (LM)	$V_{LM}\frac{dC_{LM}}{dt} = \frac{dC_{diss,LM}}{dt} + J_{LM-LF} - J_{LM-LB} - J_{LM-Drain} - \frac{dV_{LM}}{dt}C_{LM}$
Lower Fornical Sac (LF)	$V_{LF}\frac{dC_{LF}}{dt} = \frac{dC_{diss,LF}}{dt} - J_{LM-LF} - J_{LF-LP} - J_{LF-LB} - \frac{dV_{LF}}{dt}C_{LF}$
Lower Palpebral Conjunctiva (LP)	$V_{LP}\frac{dC_{LP}}{dt} = J_{LF-LP} - J_{LP-Elim}$
Lower Bulbar Conjunctiva (LB)	$V_{LB}\frac{dC_{LB}}{dt} = J_{LB-TF} + J_{LF-LB} + J_{LM-LB} - J_{LB-Elim}$
Cornea Epithelium (CorEpi)	$V_{CorEpi}\frac{dC_{CorEpi}}{dt} = J_{CorEpi-TF} - J_{CorEpi-CorStr}$
Cornea Stroma (CorStr)	$V_{CorStr}\frac{dC_{CorStr}}{dt} = J_{CorEpi-CorStr} - J_{CorStr-CorEndo}$
Cornea Endothelium (CorEndo)	$V_{CorEndo}\frac{dC_{CorEndo}}{dt} = J_{CorStr-CorEndo} - J_{CorEndo-AH}$
Aqueous Humor (AH)	$V_{AH}\frac{dC_{AH}}{dt} = J_{AH-Sec} + J_{CorEndo-AH} - J_{AH-ICB} - J_{AH-Lens} - J_{AH-VIT} - J_{AH-Drain}$
Iris-Ciliary Body (ICB)	$V_{ICB}\frac{dC_{ICB}}{dt} = J_{AH-ICB} - J_{ICB-Elim}$
Lens	$V_{Lens}\frac{dC_{Lens}}{dt} = J_{AH-Lens} - J_{Lens-VIT}$
Vitreous Body (VIT)	$V_{VIT}\frac{dC_{VIT}}{dt} = J_{Lens-VIT} + J_{AH-VIT} - J_{VIT-NFL} - J_{VIT\ (conv)}$
Retinal Nerve Fiber Layer (NFL)	$V_{NFL}\frac{dC_{NFL}}{dt} = J_{VIT-NFL} - J_{NFL-GCL} - J_{VIT-NFL(conv)}$
Retinal Ganglion Cell Layer (GCL)	$V_{GCL}\frac{dC_{GCL}}{dt} = J_{NFL-GCL} - J_{GCL-IPL} - J_{GCL-Elim} - J_{NFL-GCL(conv)}$
Retinal Inner Plexiform Layer (IPL)	$V_{IPL}\frac{dC_{IPL}}{dt} = J_{GCL-IPL} - J_{IPL-INL} - J_{IPL-Elim} - J_{GCL-IPL(conv)}$
Retinal Inner Nuclear Layer (INL)	$V_{INL}\frac{dC_{INL}}{dt} = J_{IPL-INL} - J_{INL-OPL} - J_{IPL-INL(conv)}$
Retinal Outer Plexiform Layer (OPL)	$V_{OPL}\frac{dC_{OPL}}{dt} = J_{INL-OPL} - J_{OPL-ONL} - J_{OPL-Elim} - J_{INL-OPL(conv)}$
Retinal Outer Nuclear Layer (ONL)	$V_{OPL}\frac{dC_{ONL}}{dt} = J_{OPL-ONL} - J_{ONL-PL} - J_{OPL-ONL(conv)}$
Retinal Photoreceptor Layer (PL)	$V_{PL}\frac{dC_{PL}}{dt} = J_{ONL-PL} - J_{PL-RPE} - J_{ONL-PL(conv)}$
Retinal Pigmented Epithelium (RPE)	$V_{RPE}\frac{dC_{RPE}}{dt} = J_{PL-RPE} - J_{RPE-CH} - J_{PL-RPE(conv)}$
Choroid (CH)	$V_{CH}\frac{dC_{CH}}{dt} = J_{RPE-CH} - J_{CH-SCL} - J_{CH-Elim} - J_{RPE-CH(conv)}$
Sclera (SCL)	$V_{SCL}\frac{dC_{SCL}}{dt} = J_{CH-SCL} - J_{SCL} - J_{CH-SCL(conv)}$

Table S3. Dexamethasone assumptions and calibrated parameters.

**Description**	**Parameter**	**Value**
Cornea drug permeability [m/s]	$P_{cornea}^{\#}$	2.5×10^{-8}
Conjunctiva drug permeability [m/s]	$P_{conj}^{\#}$	1.0×10^{-7}
Iris ciliary body drug permeability [m/s]	P_{ICB}^{*}	3.5×10^{-9}
Lens drug permeability [m/s]	P_{Lens}^{*}	1.0×10^{-9}
Cornea and aqueous humor drug permeability [m/s]	$P_{cor\text{-}AH}^{*}$	5.3×10^{-8}
Aqueous humor and vitreous body drug permeability [m/s]	$P_{AH\text{-}VIT}^{*}$	6.5×10^{-9}
Diffusivity of drug in the tear film regions [m/s]	D_{tf}^{*}	9.6×10^{-12}
Diffusivity of drug in the posterior eye segments [m/s]	D_{post}^{*}	4.0×10^{-5}
Elimination through the upper palpebral conjunctiva [m^3/s]	$Elim_{UP}^{*}$	4.0×10^{-15}
Elimination through the lower palpebral conjunctiva [m^3/s]	$Elim_{LP}^{*}$	4.0×10^{-15}
Elimination through the upper bulbar conjunctiva [m^3/s]	$Elim_{UB}^{*}$	4.0×10^{-15}
Elimination through the lower bulbar conjunctiva [m^3/s]	$Elim_{LB}^{*}$	4.0×10^{-15}
Elimination through the ICB [m^3/s]	$Elim_{ICB}^{*}$	6.0×10^{-15}
Elimination through the GCL [m^3/s]	$Elim_{GCL}^{*}$	5.0×10^{-15}
Elimination through the IPL [m^3/s]	$Elim_{IPL}^{*}$	5.0×10^{-15}
Elimination through the OPL [m^3/s]	$Elim_{OPL}^{*}$	5.0×10^{-15}
Elimination through the choroid [m^3/s]	$Elim_{CH}^{*}$	5.0×10^{-15}
Mixing parameter for lower meniscus	$beta_L$	1
Mixing parameter for upper meniscus	$beta_U$	1
# = calculated; * = estimated		

Table S4. Calculated and estimated ocular areas.

**Description**	**Parameter**	**Area [m^2]**
Exposed area of the cornea	$A_{cornea}^{\#}$	1.3×10^{-4}
Area of upper palpebral conjunctiva	$A_{UP}^{\#}$	5.1×10^{-4}
Area of lower palpebral conjunctiva	$A_{LP}^{\#}$	3.7×10^{-4}
Area of upper bulbar conjunctiva	$A_{UB}^{\#}$	5.3×10^{-4}
Area of lower bulbar conjunctiva	$A_{LB}^{\#}$	4.3×10^{-4}
Interfacial area between upper fornix and conjunctiva	$A_{UF}^{\#}$	1.0×10^{-3}
Interfacial area between lower fornix and conjunctiva	$A_{LF}^{\#}$	7.4×10^{-4}
Interfacial area between upper meniscus and conjunctiva	$A_{UM}^{\#}$	2.1×10^{-5}
Interfacial area between lower meniscus and conjunctiva	$A_{LM}^{\#}$	6.8×10^{-5}
Interfacial area between AH and ICB	A_{ICB*}	7.0×10^{-4}
Interfacial area between AH and lens	A_{Lens*}	6.2×10^{-7}
Retinal surface area	$A_{Retina*}$	1.2×10^{-3}
# = calculated; * = estimated		
